# Three People Can Synchronize as Coupled Oscillators during Sports Activities

**DOI:** 10.1371/journal.pcbi.1002181

**Published:** 2011-10-06

**Authors:** Keiko Yokoyama, Yuji Yamamoto

**Affiliations:** 1 Graduate School of Education and Human Development, Nagoya University, Furo-cho, Chikusa, Nagoya, Japan; 2 Japan Society for the Promotion of Science, 5-3-1, Koujimachi, Chiyoda-ku, Tokyo, Japan; 3 Research Center of Health, Physical Fitness and Sports, Nagoya University, Furo-cho, Chikusa, Nagoya, Japan; University College London, United Kingdom

## Abstract

We experimentally investigated the synchronized patterns of three people during sports activities and found that the activity corresponded to spatiotemporal patterns in rings of coupled biological oscillators derived from symmetric Hopf bifurcation theory, which is based on group theory. This theory can provide catalogs of possible generic spatiotemporal patterns irrespective of their internal models. Instead, they are simply based on the geometrical symmetries of the systems. We predicted the synchronization patterns of rings of three coupled oscillators as trajectories on the phase plane. The interactions among three people during a 3 vs. 1 ball possession task were plotted on the phase plane. We then demonstrated that two patterns conformed to two of the three patterns predicted by the theory. One of these patterns was a rotation pattern (*R*) in which phase differences between adjacent oscillators were almost 2π/3. The other was a partial anti-phase pattern (*PA*) in which the two oscillators were anti-phase and the third oscillator frequency was dead. These results suggested that symmetric Hopf bifurcation theory could be used to understand synchronization phenomena among three people who communicate via perceptual information, not just physically connected systems such as slime molds, chemical reactions, and animal gaits. In addition, the skill level in human synchronization may play the role of the bifurcation parameter.

## Author Summary

Synchronization is very interesting as both a natural phenomenon and scientific topic in physical and biological systems. Examples include the Belousov-Zhabotinsky (BZ) reaction, the oscillation of metronomes, the flash of fireflies, and the calling behavior of Japanese tree frogs. The symmetric Hopf bifurcation theory, which is based on group theory, has been proposed as a useful approach for spatiotemporal pattern formation in coupled oscillator systems. This theory has been applied to various types of quadrupedal gaits in terms of symmetrically coupled oscillators, and to rings and chains of coupled oscillators of plasmodial slime molds. Here we report that the spatiotemporal pattern formation in three-person coupling during dynamic human movement, such as sports activity, conforms to symmetry-breaking theory. Our present study is salient because the spatiotemporal synchronization patterns among three people corresponded to the predicted patterns derived from symmetric Hopf bifurcation theory, as with pattern formation in slime molds, even though the actors were not connected physically but informationally. Moreover, although informational coupling between two people has been shown previously in well controlled experiments, we demonstrate three-person coupling using perceptual information in a real-life setting.

## Introduction

Synchronization has been both experimentally and theoretically analyzed in many biological systems from micro- to macroscales [1], [2]. In particular, human synchronization has focused on interactions between two people or among a large group [3]–[7]. Surprisingly, however, small groups of three to four people have never been analyzed. We experimentally analyzed the interaction among three people to clarify the synchronization patterns generated by a small group.

Many studies focusing on human synchronization have used equations for coupled nonlinear oscillators based on phase models [6]–[10]. In these studies, the behavior of one person was treated as a nonlinear oscillator, and interactions between people were formulated as interactions among nonlinear oscillators. For example, the behavior of two people swinging their legs while watching each other has been studied using a model of two coupled oscillators and two synchronization patterns in-phase and anti-phase. It was thought that these patterns are generated by the visual interaction between the two people [6]. This mathematical model, however, requires detailed parameters such as the natural frequency of each oscillator and coupling strength between the oscillators. Hence to apply this model to natural phenomena, we must manipulate and measure these parameters. To avoid this methodological problem, many studies on two-person synchronization have analyzed cyclic movements using metronomes to manipulate the oscillator frequency and measured the leg and pendulum displacement in situations unrelated to daily life [6]–[10]. These methodological problems are more complex when the model is extended from two to three coupled oscillators. In other words, the equation of the model of three coupled oscillators would be more complicated than that of two coupled oscillators. Also, manipulating and measuring parameters experimentally in this case would be more difficult. Although synchronization of three frogs has been mathematically simulated[11], studies applied to models of three coupled oscillators have not been analyzed experimentally.

However, the Kuramoto model of coupled oscillators based on phase equations that include simple mathematical models has described the interactions among many oscillators. This model describes many synchronized oscillators in large groups by one equation using the mean field approximation[12]. It does not require the measurement of many parameters experimentally because it can describe phenomena via one collective parameter. Using this framework, collective clapping in concert halls has been shown to exhibit synchronized and unsynchronized patterns repeatedly[3], [4]. In summary, models of coupled oscillators based on phase equations are not appropriate for analyzing small groups experimentally, because the numbers of oscillators in small groups are too large to solve the equations and to identify the intrinsic dynamics. On the other hand, those numbers are too small to use the mean field approximation that is appropriate in large groups.

However, symmetric Hopf bifurcation group theory is useful for analyzing synchronization in a small group. Although this theory is based on coupled oscillators, it can provide some spatiotemporal patterns from only geometrical symmetry, even if the intrinsic parameters in phenomena such as the frequency and coupling strength cannot be manipulated and measured experimentally[13]. For example, some gait patterns of multi-legged animals generated by central pattern generators have been found to correspond to patterns predicted by symmetric Hopf bifurcation theory when certain geometrical symmetries are taken into account [14]–[20]. These studies showed that the symmetry included in natural phenomena could be understood in strict mathematical terms. It also suggests that this framework is appropriate for complex phenomena for which capturing the individual elements is difficult because we can predict the spatiotemporal patterns generated by the geometrical symmetry using symmetry breaking before analyzing the experimental data. In the spatiotemporal patterns in rings of three-, four-, and five-oscillator systems of plasmodial slime molds that did not know the intrinsic dynamics, all types of oscillation mode were demonstrated from mathematical formulae by patterning of cell shapes using microfabricated structures[21]. Chains of coupled oscillators have also shown the spatiotemporal patterns predicted by this theory[22]. However, hidden symmetric patterns that were not derived from the explicit geometry of the system for chains of three coupled oscillators were also found in the same study. Hence the symmetric Hopf bifurcation group theory provides a list of possible synchronized patterns generated by geometric forms, even if the intrinsic dynamics of the complex individual oscillators cannot be found from a conventional phase equation analysis.

**Figure 1:**
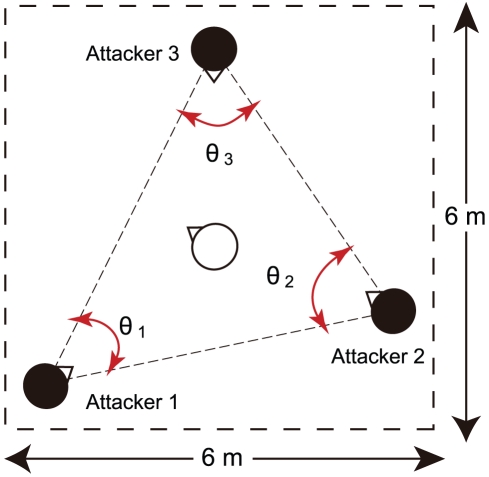
The experimental task: a 3 vs. 1 ball possession task in soccer practice for studying interactions among three people. Three attackers (Attackers 1, 2, 3) were asked to pass a ball as much as possible, without allowing it to be intercepted by one defender for 90 s in a 6-m square. Angular oscillations were constructed by the three attackers (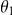, 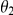, 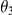).

In this study, we experimentally analyzed a human movement task with geometric symmetry to clarify the synchronization among three people using symmetric Hopf bifurcation theory. The formation of symmetry in coupled oscillators requires that all coupling strengths between the oscillators be identical. Therefore, finding a task was necessary in which the interactions between people were equal. We focused on a sports activity that could bring out natural synchronization: 3 vs. 1 ball possession in soccer practice[23]. This demands the cooperative behavior of passing a ball among three attackers, while keeping the ball from one defender (Figure 1). The three attackers in this task have to move in a restricted area to interact with each other and pass as much as possible. Because each pair among the three attackers could interact and pass a ball to the other attacker, this task was regarded as the formation of a ring of coupled oscillators. We investigated the angle oscillations between the three attackers in a ring of three oscillators with neighbor coupling. In addition, we regarded the interaction strengths as equal when the skill levels of the three attackers were equal. Hence synchronization among three people in a 3 vs. 1 ball possession task should show the spatiotemporal patterns predicted by symmetric Hopf bifurcation theory in a ring of three coupled oscillators with geometric symmetry.

## Materials and Methods

### Predicted three synchronization patterns on the phase plane

**Figure 2:**
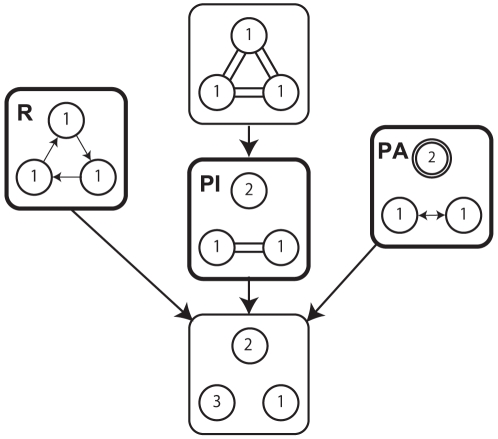
Five predicted patterns of a ring of three coupled oscillators from symmetric Hopf bifurcation theory. The number in the circle indicates the number of the waveforms. The double circle shows that the corresponding oscillator has double frequency. Relationships between two oscillators are indicated by  = : in-phase, 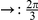 phase shift, 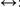 anti-phase.

Symmetric Hopf bifurcation theory can create synchronization patterns for, e.g., three-, four-, and five-oscillator systems[13]. Analysis by this theory requires only geometric symmetry, not the intrinsic dynamics of the system or the nature of the coupling between oscillators. That is, the theory considers a network of *n* coupled identical oscillators together in ring geometry with symmetry and identical couplings. For example, a network of three identical oscillators is coupled together in a ‘triangle’ formation. This symmetric formation can provide two different types of symmetry, spatial and temporal, which leads to spatiotemporal patterns in the system. In other words, oscillation patterns generated from a system can be provided only from a spatially symmetric formation of the system. These patterns correspond to a different isotropy subgroup of 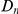 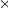 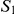. The dihedral group 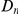 is the symmetry of a regular *n*-gon. Five patterns are predicted from symmetric Hopf bifurcation theory for the three coupled oscillators treated in this study (Figure 2). The first pattern is an all in-phase pattern, in which all three oscillators have identical waveforms and move in phase. It maintains both spatial and temporal symmetry perfectly. The second pattern is a rotation pattern, in which all oscillators have identical waveforms but are phase-shifted by 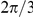. The third pattern is a partial anti-phase pattern in which two of the oscillators have identical waveforms but are phase-shifted by *π*; the third oscillator has a different waveform and twice the frequency of the other two. The fourth pattern is a partial in-phase pattern in which two of the oscillators behave identically (i.e., they have the same waveform and move in phase) and the third oscillator has a different waveform. The fifth pattern is a different pattern in which all three oscillators have a different waveform. This last pattern is the most asymmetric. It results from symmetry breaking in a system of coupled identical oscillators.

**Figure 3:**
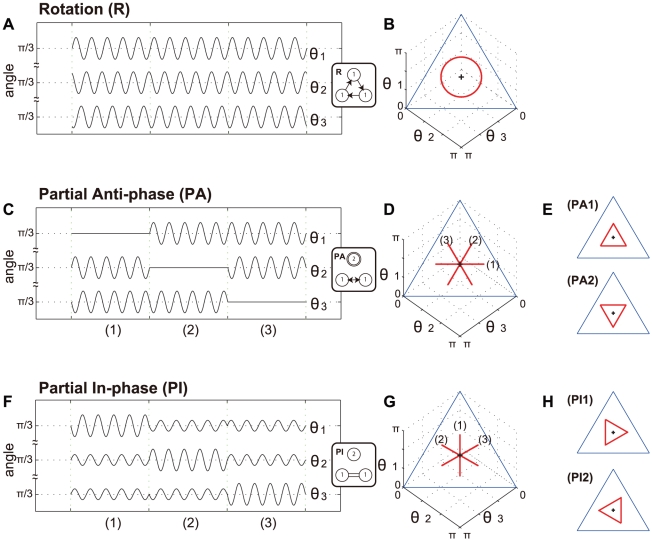
Three synchronized patterns in a 3 vs. 1 ball possession task as rings of three coupled oscillators predicted by symmetric Hopf bifurcation theory. (A, C, F) Time series of angles. (B, D, G, E, H) Trajectories on the phase plane. (B), (D), and (G) show the phase plane of time series in (A), (C), and (F), respectively. (+) in the phase plane shows that all three oscillators would have 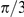 equally. (A, B) A rotation pattern (*R*) in which all three oscillators are synchronized while keeping the phase difference 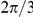 . (C-E) A partial anti-phase pattern (PA) in which two oscillators are synchronized in anti-phase and another is constant. PA1 shows the case in which the constant value is smaller than 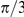 and the value of PA2 is larger than that of 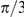. (F-H) A partial in-phase pattern (*PI*) in which two oscillators are synchronized in-phase and another is in anti-phase synchronization. 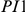 and 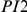 show the cases in which one of the two in-phase oscillators is larger than 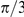 and the other one is smaller than 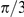 and vise versa.

Rotation, partial in-phase, and partial anti-phase patterns were found in three-coupled biological oscillators of slime molds[21]. We assume that three-person coupled oscillators would also exhibit these patterns. Figure 3 shows simplified models for these three synchronization patterns. Figures 3A, 3C, and 3F show time series of oscillations for the angles constructed between each pair of the three attackers, as shown in Figure 1. These three angle oscillations are plotted as trajectories in three-dimensional (3-D) space, as shown in Figures 3B, 3D, and 3G. Because the sum of the three angles 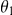, 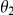, and 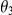 is always *π*, these trajectories could be drawn on the two-dimensional (2-D) plane. Each vertex on the phase plane represents the case in which one oscillator has angle *π*, and the other two oscillators have zero degrees. The center of this 2-D phase plane (+) represents the case where all three oscillators have 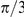 equally. This phase plane can express the relationships among three oscillators as a geometric trajectory; that is, an attractor showing the system behavior. This allows us to compare the predictive patterns visually with experimental data.

Figures 3A and 3B show a rotation pattern whereby all three oscillators are synchronized with a constant phase difference of 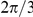. This pattern takes a circular trajectory, centered on the phase plane (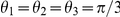). The amplitudes of all oscillators correspond to the radius of the circle (Video S1). Figures 3C-E show a partial anti-phase pattern. In this case, two oscillators are in anti-phase synchronization, while the other is in a half-period oscillation. This interesting feature was reported in slime molds[21]. In this study, we did not hypothesize the features of a half period, and defined one oscillator as constant in the time series (Video S2). This solution was derived from the fact that the total of the amplitudes of all oscillators was *π*. The conditions of a partial anti-phase pattern suggested by symmetric Hopf bifurcation theory were that ‘two of the oscillators have identical waveforms’ and ‘no oscillators are in-phase with each other’[13]. Therefore, the oscillator without identical waveforms to the other two remains constant. This pattern is called the ‘death anti-phase pattern’ in chemical oscillator systems such as the Belousov-Zhabotinsky (BZ) reaction[24]. In Figure 3D, we show that the constant oscillator was switching in the order of 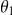, 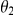, and 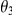. For example, in phase (1), 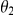 and 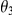 were anti-phase, and 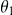 was constant at 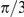. The trajectory on the phase plane moved parallel to the edge and passed the center with the corresponding amplitude. In the case of a constant oscillator without phase 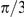, the trajectory would be parallel to the edge of the phase plane, but shift up and down corresponding to that value. 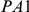 is the case when the constant value is smaller than 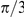, while 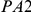 is that when the value is larger than 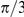 in Figure 3E.

Figures 3F-H show a partial in-phase pattern when two oscillators are synchronized in-phase and the other oscillates. In this pattern, two oscillators have the same waveform and are in-phase. For example, the case of phase (1) shows that 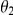 and 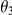 are in-phase. The other oscillator is in anti-phase synchronization at twice the amplitude because the total amplitude of the three oscillators is *π*. In this pattern, the phase trajectory is parallel to perpendicular on the phase plane. Moreover, all trajectories need not pass through the center of the phase plane. 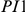 and 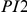 show examples in which one of the two in-phase oscillators is larger than 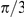 and the other one is smaller than 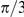 (Figure 3H).

### Participants

In total 48 subjects (16 females and 32 males; 19–21 years old) provided written informed consent prior to the experiment and were included in the study. Procedures were approved by the Internal Review Board at the Research Center of Health, Fitness, and Sport at Nagoya University and conformed to the principles expressed in the Declaration of Helsinki. A group of 16 high-level players, 16 mid-level players, and 16 low-level players participated in the experiment. The high- and mid-level groups were male soccer players from a university team that was at the national level in Japan. The high-level players were in the starting lineups for the top team, while the mid-level players were not. The low-level group consisted of female futsal players who had not played football or soccer before they entered the university.

### Procedure

Each group was divided into four subgroups of four players and played a 3 vs. 1 ball possession task for 90 s in each trial, with four trials per subgroup. The number of trials in each subgroup was equal, such that all participants played once defensively. We asked the three attackers to keep possession from the defender and pass the ball to other attackers as much as possible within a 6-m square, and the defender to make interceptions whenever possible (Video S3). The mean number of continuous passes for each group was tested by one-way ANOVA. A significant difference was observed, with 13.88±2.75, 8.76±2.55, and 4.89±1.49 for the ‘high-,’ ‘mid-,’ and ‘low-’ level groups, respectively (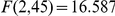, *p*<0.001). Multiple comparisons using Tukey's post hoc test suggested that all pairs between the two groups had significant differences: high-middle: *p*<0.01, high-low: *p*<0.001, and middle-low: *p*<0.05. Because the higher-level groups could connect more passes than the lower-level ones, significant differences among the three groups were observed in their ball possession skills.

The experiment was recorded with a video camera (Sony HDR-XR550V) operating at 30 frames per second, which was placed high-up so that the whole area in the experiment could be recorded. The direct linear transformation (DLT) method for 2-D reconstruction from images was used to retrieve position data in 2-D coordinates for the attackers. The coordinates of the players were captured from the centers of their heads. To apply the DLT method, twelve control points were positioned at 1.5-m intervals around a 6-m square. These control points were recorded as the centers of the experimenter's heads when they were upright with almost the same height as the participants. The mean errors in reconstructing the control points were 2.3 and 4.6 cm in the high- and mid-level groups, and 1.3 and 2.1 cm in the low-level groups, for the *X*- and *Y*-axes, respectively.

### Data analysis

The coordinates of the three points representing the attackers in the 2-D image were digitized in 30 Hz. These data were reconstructed in 2-D using Frame-DIAS (DKH). High-frequency noise was reduced using a second-order Butterworth digital filter, with 1.0 and 0.5 Hz used for the *X*- and *Y*-axes, respectively. The time series data of the three angles were calculated from the triangle that was constructed from the three attackers' coordinates. Data during interception by the defender were excluded.

## Results

**Figure 4:**
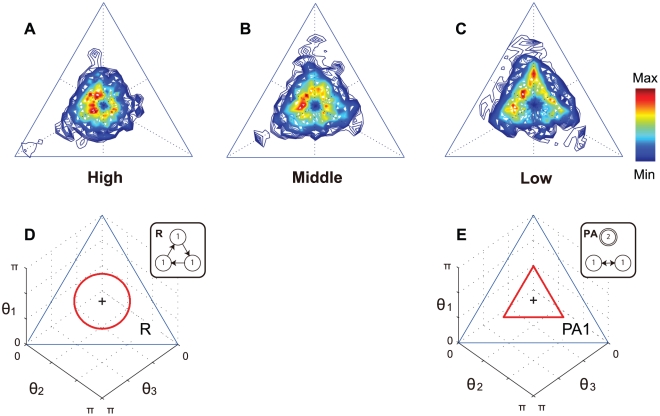
Comparison between the experimental data and predicted patterns. (**A-C**) Color contour plots show experimental time-frequency trajectory plots on the phase plane. (**A**), (**B**), and (**C**) show the high-, mid-, and low-level groups, respectively. The color indicates the height normalized by the maximum and minimum frequency values for each skill level. Dark red is the highest and dark blue is the lowest, while white represents no trajectories. (**D, E**) Two predicted trajectories on the phase plane selected from Figure 2. (**D**) shows a rotation pattern (*R*) that is similar to (**A**) for the distribution of the high-level group. (**E**) shows a partial anti-phase pattern (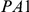) that is similar to (**B**) and (**C**) for the mid and low levels, respectively.

The time series of the angle data for each skill level were superimposed as trajectories on the phase plane (Figure S1). Based on these trajectories, to capture the dominant pattern of each level, we show color contour plots in the phase plane that represent the time frequency of trajectories normalized by the maximum and minimum values in each skill level, as shown in Figures 4A-C. The dark red (blue) shows the highest (lowest) frequency of the trajectories, and the area not painted indicates no trajectory. The distribution of trajectories for the high-level group (Figures 4A) shows a circular pattern based on the center of the phase plane. In other words, all three angles are around 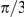. In contrast with the distribution for the high-skill group, the distribution for the low-level group spread to the vertex and parallel to the edge of the phase plane (Figure 4C). The distributions for the mid-level group show an intermediate pattern between the high- and low-level groups (Figure 4B). Figures 4D and 4E show a rotation (*R*) and partial anti-phase pattern (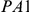) from a symmetric Hopf bifurcation theory (Figure 3). This suggests that the distribution for the high-level group corresponds to a rotation pattern (*R*), while the mid- and low-level groups correspond to a partial anti-phase pattern in which the two oscillators are in anti-phase synchronization and the other is constant. When the constant angle is smaller than 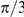, the distribution patterns for the mid and low levels correspond to the predicted pattern 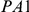.

**Figure 5:**
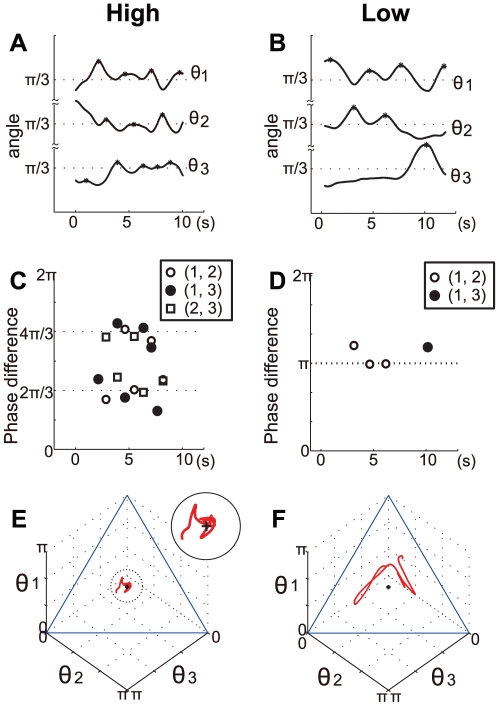
Typical examples of the high- and low-skill-level over 10 s. (**A, B**) Time series of three angle oscillations. The scale of angle A was reduced so that the scale in (**A**) could be three times smaller than that in (**B**). (*) in the time series indicates peaks. (**C, B**) Phase difference between any two angles. These values were calculated from the peaks in (**A**) and (**B**). (**E, F**) Trajectories on the phase plot for the above time series.

To examine whether the observed distribution patterns of the trajectories corresponded to the predicted patterns, the phase differences were calculated using the inflection points on the time series (text S1, Figure S2, S3, S4). Based on the time series analysis, the most frequent pattern in the high-level group was rotation (16.6%), followed by partial anti-phase (12.7%) and partial in-phase (6.8%). In contrast, the most frequent pattern in the low-level group was partial anti-phase (14.5%) followed by rotation (13.0%) and partial in-phase (9.6%). Two kinds of permutation tests using occurrence frequencies and subgroups showed significant differences between the two groups for the three patterns (occurrence frequencies: *p* = 0.018, subgroups: *p* = 0.058) (text S2). The time series of the angles during 10 s for the high and low levels are shown in Figures 5A and 5B as typical examples. Figures 5C and 5D show the phase differences calculated using the peaks between two angles, while Figures 5E and 5F represent the trajectory on the phase plane. In the high-level group, the values of the phase differences are near 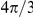 or 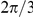. This suggests a rotation pattern, in which the three angles are synchronized, maintaining a 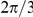 phase difference but changing their order. However, the phase differences between two angles of the low-level group were around *π* (i.e., anti-phase), with the other angle almost constant. This feature was a partial anti-phase pattern. However, the trajectory on the phase plane did not pass the center because the asynchronous angle was constant and smaller than 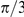. As Figure 5F shows, the trajectory moved parallel to the edge of the phase plane during first 8 s and then changed direction to the other edge. This means that the angles 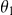 and 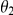 synchronized in anti-phase and the angle 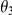 was constant with value smaller than 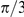 during the first eight seconds. Then, angles 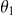 and 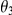 reached anti-phase synchronization and 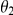 became constant. The color contour plots in Figure 4 show the time frequency of these trajectories.

## Discussion

We analyzed a natural and goal-directed human movement task with geometrical symmetry, 3 vs. 1 ball possession, by applying symmetric Hopf bifurcation theory[13]. Five patterns were predicted from this theory for a symmetric ring network of three coupled oscillators. Three of these patterns were reported previously in slime molds[21] and depicted on the phase plane in 3-D oscillator space. We defined the following three patterns: rotation (*R*), partial anti-phase (*PA*), and partial in-phase (*PI*). The rotation pattern occurred when all three oscillators were synchronized with the phase difference 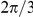. The partial anti-phase pattern (*PA*) occurred when two oscillators were in anti-phase synchronization and the other was constant. The partial in-phase pattern (*PI*) occurred when two oscillators were in in-phase synchronization and the other was oscillating. The differences in these patterns were clearly described as the attractors of trajectories on the phase plane. The attractors were circular for *R*, parallel to the edge of the phase plane for *PA*, and perpendicular to the edge of the phase plane for *PI*. Although time series analysis revealed not only *R* and *PA* patterns but also *PI* and other patterns (Table S1), this analysis was based on the combination of relationships between two oscillators, and did not describe the behavior among the three oscillators as a system directly. Thus, we developed an analysis method using the phase plane to depict the feature of synchronization among three oscillators as attractors. When the observed angle data of the high-, mid- and low-skill-level groups were plotted on the phase plane, it suggested that the high- and low-skill-level groups corresponded to *R* and 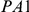, respectively.

The two patterns *R* and *PA* have also been observed at the microscale of a synchronized phenomenon, i.e., biological oscillators in slime molds[21]. Although the dynamics of slime molds and people as oscillators are obviously different, common synchronized patterns were revealed in both the micro- and macroscale phenomena. This strongly suggests that symmetric Hopf bifurcation theory could provide a list of possible patterns from the geometrical symmetry of coupled oscillators, which does not depend on the intrinsic dynamics of oscillators.

Note that in the present study, three attackers were not connected by a material link like the slime mold[21] or a central pattern generator[15]–[20], [25]. Synchronization was achieved via a goal-oriented human movement task that required the three attackers to keep a ball while avoiding interception by a defender. Previous research on synchronization between two people showed that the same principles underlie within- and between-person interaction using only optical information[6]. Hence, we consider that in the present study, three attackers or oscillators could also be connected by optical or visual information. From the results, two important findings were revealed. First, symmetric Hopf bifurcation theory can explain phenomena connected by both physical and perceptual information. Second, humans coupled by perceptual information can synchronize between both two and three people.

In this respect, the experimentally observed patterns in different skill groups show different synchronization patterns. Groups of high- and low-skill-levels suggest rotation and partial anti-phase patterns, respectively. In a rotation pattern (*R*), all three oscillators are synchronized while the phase difference is constant at 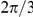. This synchronization pattern requires coupling between each pair among the three oscillators. In partial anti-phase and in-phase patterns, two oscillators are in anti-phase and in-phase synchronization, and the other one is constant (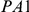) and in anti-phase synchronization (*PI*), respectively. These patterns need only one coupling among the three oscillators. Together with the informational coupling in this task, this result suggests that a high-skill-level group is connected informationally in three ways, while a low-skill-level group is connected in only two ways; i.e., in the high-skill-level group every attacker was connected to two other attackers, whereas in the low-skill-level group, every attacker was connected to one other attacker. The difference in synchronization pattern is likely caused by the difference in skill level. In other words, we might be able to regard the skill level as a bifurcation parameter of human synchronization in this movement task.

However, we should bear in mind that these results are a dominant pattern based on the time frequency on the phase plane at each skill level. Spontaneous switching behavior among some synchronized patterns was observed in slime molds, in which the behavior depended on the coupling strength controlled by the channel width between oscillators[26]. Accordingly, if we analyze time series within each trial in detail, we might be able to find switching between synchronized patterns. In addition, task constraints would affect the pattern formation of synchronization. For example, a movable area would affect the amplitude of the oscillations, and the relative skill level between attackers and defender would influence the coupling strength among the oscillators as an environmental or a bifurcation parameter in the system. To examine different couplings, such as those where all three oscillators have identical waveforms and move in phase, we could manipulate these experimental variables. To better understand the dynamics of human synchronization, we must investigate the relationship between the patterns shown in this study and switching behavior or various parameters.

In conclusion, we demonstrated that human synchronization among three people can be understood as a ring of three coupled oscillators, similar to slime molds, as based on symmetric Hopf bifurcation theory; humans synchronize using informational, not material, linkage; and the skill level in human movement plays the role of the bifurcation parameter for synchronization pattern formation. Symmetric Hopf bifurcation theory would be useful for understanding complex/macroscale human synchronization because it provides possible patterns from only geometrical symmetry, without requiring knowledge of the intrinsic dynamics of the system [26]. That is, this model-independent approach [13] may help to clarify complex human movement patterns in natural situations related to daily life, such as sports activity. This can be done without manipulating or measuring detailed parameters such as the natural frequency of each oscillator or the coupling strength between oscillators. We will be able to extend this approach to larger systems, such as 4 versus 2 and 5 versus 3 games, based on four or five coupled oscillators. Moreover, ball games, such as handball, football, or hockey, including geometrical symmetry might be studied via this approach, because these games have strict rule constraints, such as the number of players or the area of playing fields. However, we need to examine in detail the collective variables describing the system behavior. To provide stronger evidence of the applications of symmetric Hopf bifurcation theory, we must analyze more small groups, such as those of four or five people, and compare them with the predictive patterns in the theory. Finally, a dynamic system perspective, including symmetric Hopf bifurcation theory, may be able to bridge the gap between theory and practice[27], [28] to design a coaching philosophy and a practical training regimen[29].

## Supporting Information

Figure S1 **Examples of time series of three groups for 90 s in one trial.** (**A, B, and C**) Time series of three angle oscillations for high-, mid-, and low-level groups, respectively. The bars at the bottom of the time series show the duration of ball possession, and the blanks between time series indicate excluded data due to defender interception. (**D, E and F**) Trajectories on phase plane for each time series corresponding to **A, B, and C**.
(PDF)

Figure S2
**Schematic classification diagram of the phase difference between two oscillators.** (**A**) in-phase, (**B**) anti-phase, (**C**) 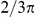, (**D**) ‘death’ pattern of the phase difference we defined.(EPS)

Figure S3
**Examples of classification of synchronized patterns among three oscillators.** Black lines indicate the time series of angle, and (+) shows the inflection point of those lines. The color bars around each time series of angle show the different synchronized patterns among the three oscillators, *R*, *PA*, and 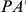, which were calculated by the reference interval between inflection points (a peak and a valley) of each time series of that angle. These patterns (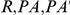) are shown by red, blue, and green bars, respectively. The bars shown on top of the three time series were finally defined as the synchronized pattern among the three oscillators.(EPS)

Figure S4 **Examples of time series of high- and low-level groups and switching patterns.** (**A**) Time series of three angle oscillations for high-level group. (**B**) Time series of three angle oscillations for low-level group.
(EPS)

Table S1 Observed duration of each pattern for high- and low-level groups.
(PDF)

Text S1 Time series data analysis.
(PDF)

Text S2 Permutation test.
(PDF)

Video S1 Animated clip showing the pattern of movement of players on the field, and the time series of three angles for each of the ideal rotation (*R*) oscillatory modes.
(AVI)

Video S2 Animated clip showing the pattern of movement of players on the field, and the time series of three angles for each of the ideal partial anti-phase (*PA*) oscillatory modes.
(AVI)

Video S3 Real movie showing an example of the pattern of movements of high-level players on the field.
(WMV)
