# Supplementary figures and images for: Three People Can Synchronize as Coupled Oscillators during Sports Activities
Source: PLoS Comput Biol. 2011 Oct 6;7(10):e1002181. doi: 10.1371/journal.pcbi.1002181 (PMC3188505; doi:10.1371/journal.pcbi.1002181)

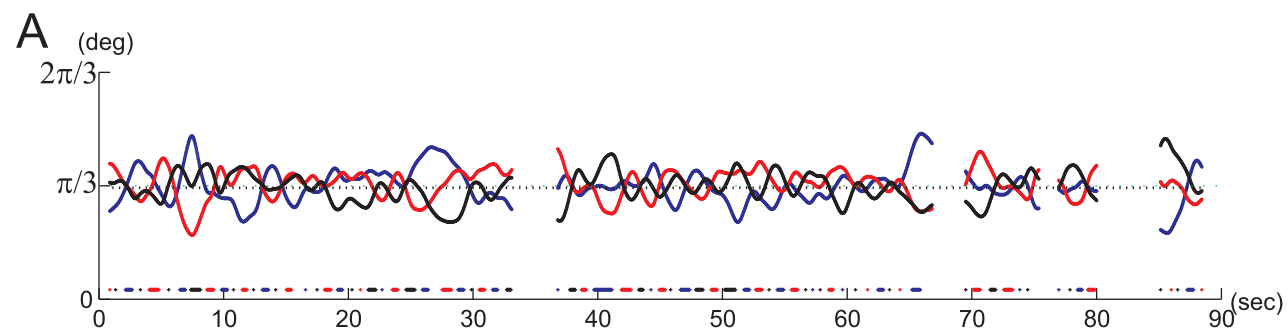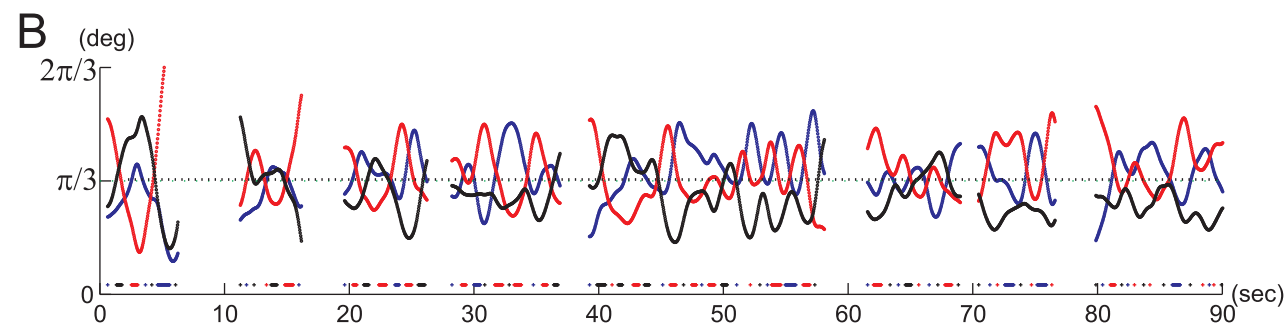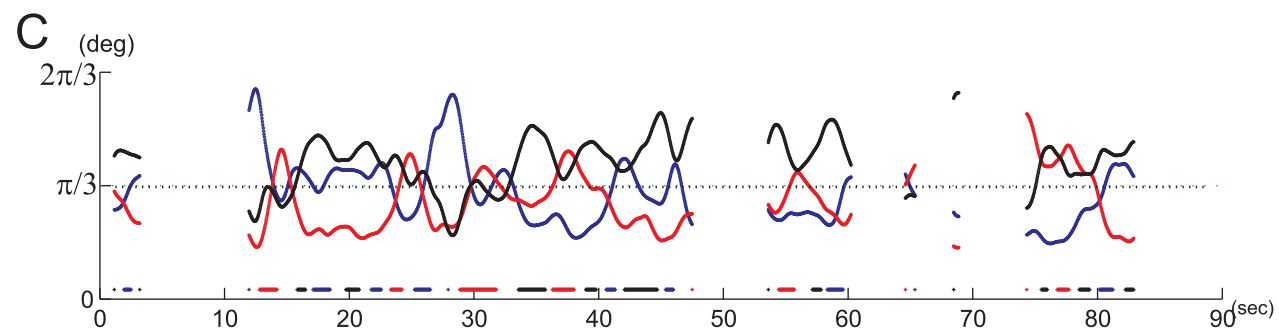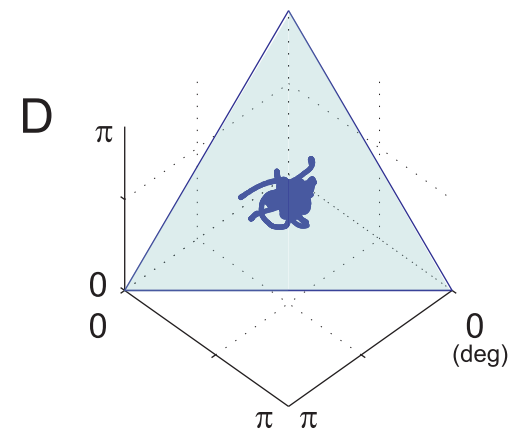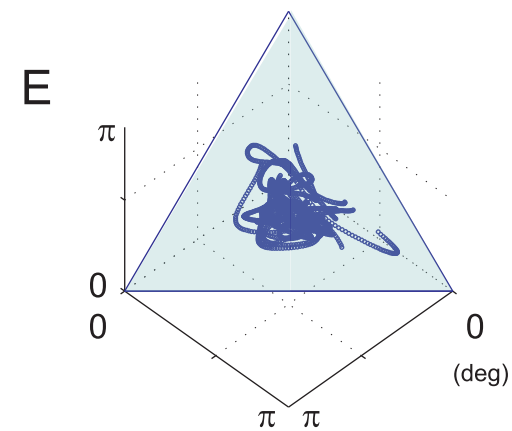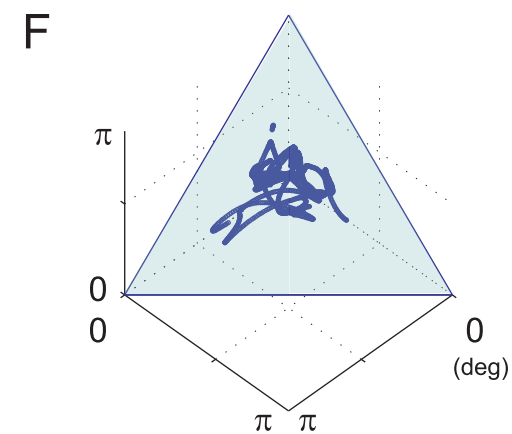

Supplement: Figure S1 — Examples of time series of three groups for 90 s in one trial. (A, B, and C) Time series of three angle oscillations for high-, mid-, and low-level groups, respectively. The bars at the bottom of the time series show the duration of ball possession, and the blanks between time series indicate excluded data due to defender interception. (D, E and F) Trajectories on phase plane for each time series corresponding to A, B, and C. (PDF) [file pcbi.1002181.s001.pdf]
