# Supplementary material for: Three People Can Synchronize as Coupled Oscillators during Sports Activities
Source: PLoS Comput Biol. 2011 Oct 6;7(10):e1002181. doi: 10.1371/journal.pcbi.1002181 (PMC3188505; doi:10.1371/journal.pcbi.1002181)
Supplement: Table S1 — Observed duration of each pattern for high- and low-level groups. (PDF) [file pcbi.1002181.s005.pdf]

Table S1

Table 1:

Pattern	R	PA	PI	PA'	PI'	Other	Total
High	162.2	124.4	66.5	340.2	2.5	280.6	976.4
Low	68.1	75.7	50.3	170.0	2.8	156.3	523.1

(sec)
