# Supplementary material for: Three People Can Synchronize as Coupled Oscillators during Sports Activities
Source: PLoS Comput Biol. 2011 Oct 6;7(10):e1002181. doi: 10.1371/journal.pcbi.1002181 (PMC3188505; doi:10.1371/journal.pcbi.1002181)
Supplement: Text S1 — Time series data analysis. (PDF) [file pcbi.1002181.s006.pdf]

Text S1

Time Series Data Analysis

Time courses of angle change in each trial were calculated from the triangle that consisted of the three attackers' coordinates (see Fig. 1). Typical time courses for the high-level and low-level groups are shown in Figure S1. In the ideal and noise-free cases, the phase and/or frequency were generally estimated by the time interval between two peaks corresponding to one complete cycle. In the present study, the waveforms were changed suddenly, and there was not a complete cycle from one peak to the next. This was because the intermittent external force (the defender's movement) added to the ring of the three coupled oscillator system. The series of peaks and valleys for each oscillator, θ_i can be considered a sequence of point events taking place at time $t_k, k = 1, 2, \ldots$. The time interval between two inflection points (a peak and a valley) corresponds to one half cycle, π, as a reference interval, and for an arbitrary instant of time, t, for other each oscillator θ_j, as a comparator to θ_i, $t_k < t < t_{k+1}$ determines the phase as a linear interpolation between these values.

$$\phi(t) \quad = \quad \pi \frac{t - t_k}{t_{k+1} - t_k}$$

At the first step, we detected the inflection points for each oscillator, the inflections were defined as angle changes of more than 2% of the variance in each trial. Next, we calculated phase differences for all pairs between two oscillators, $(\theta_i,\, \theta_j) = (\theta_1,\, \theta_2)$, $(\theta_1,\, \theta_3)$, $(\theta_2,\, \theta_1)$, $(\theta_2,\, \theta_3)$, $(\theta_3,\, \theta_1)$, $(\theta_3,\, \theta_2)$, based on the locations using the equations above. In the next step, phase relations between two oscillators, such as in-phase, anti-phase, and $2/3\pi$ rotation, were defined when the phase differences were at $0 \pm 1/6\pi$, $\pi \pm 1/6\pi$, and $2/3\pi \pm 1/6\pi$, respectively. In this step, when there was no inflection point as a comparator in a certain reference interval, we categorized it as a 'death' (see Figure S2). Also, when there were more than two inflections as comparators in a certain reference interval, we categorized it as another pattern.

The oscillations were categorized into six patterns; *R*, *PA*, *PI*, *PA'*, *PI'*, and *O*. In rotation mode (*R*), the phase differences between adjacent oscillators were almost $2/3\pi$. When two phase differences showed $2/3\pi$ simultaneously, the pattern was classified into pattern *R*. In partial anti-phase mode (*PA*), two oscillators showed anti-phase and the third oscillator showed a frequency doubling oscillation [1] or "amplitude death" [2]. In this study, when one of the phase differences was anti-phase and the third oscillator had no peak or valley, the pattern was classified as *PA*. In partial in-phase mode (*PI*), two oscillators were in-phase and the third oscillator was anti-phase. When one of the three phase differences was in-phase and the others were anti-phase, the pattern was classified as *PI*. Other oscillation patterns with lower spatio-temporal symmetry were classified into *PA'* and *PI'*, based on a previous study [3]. In pattern *PA'*, two oscillators were anti-phase and the other was independent of the two oscillators.

In pattern *PI'*, two oscillators were in-phase and the other was independent of the two oscillators. When all combinations of oscillators showed desynchronization, such cases were classified as other (*O*). These patterns were categorized from the beginning to the end of each trial. The pattern was searched in the order of *R*, *PA*, *PI*, *PA'*, and *PI'*, according to the difficulty in synchronization. When the same pattern overlapped continuously among pairs, the durations were connected with the same pattern. When the next different pattern started from the duration of the current pattern, the duration was counted as a remaining duration. These procedures were programmed for the analysis using MATLAB. Examples of these classifications in time series for the high- and low-level groups are shown in Figures S3 and S4.

References

1. Takamatsu A, Tanaka R, Yamada H, Nakagaki T, Fujii T, et al. (2001) Spatiotemporal symmetry in rings of coupled biological oscillators of physarum plasmodial slime mold. Physical Review Letters 87: 078102.

2. Yoshimoto M, Yoshikawa K, Mori Y (1993) Coupling among three chemical oscillators: synchronization, phase death, and frustration. Physical Review E 47: 864-874.

3. Takamatsu A (2006) Spontaneous switching among multiple spatio-temporal patterns in three-oscillator systems constructed with oscillatory cells of true slime mold. Physica D 223: 180-188.
