# Supplementary material for: Three People Can Synchronize as Coupled Oscillators during Sports Activities
Source: PLoS Comput Biol. 2011 Oct 6;7(10):e1002181. doi: 10.1371/journal.pcbi.1002181 (PMC3188505; doi:10.1371/journal.pcbi.1002181)
Supplement: Text S2 — Permutation test. (PDF) [file pcbi.1002181.s007.pdf]

Text S2

Permutation test

We applied a permutation test [1] to the results of the time series analysis for the observed duration of each pattern, for high- and low-level groups (see Table S1). We calculated the C-value representing the difference between observed and expected data as follows:

$$C = \sum \frac{(O - E)^2}{E}$$

O and E denote observed and expected data, respectively.

Here, we focused on the difference between high- and low-level groups only for R, PA, and PI patterns, because the others could be regarded as incomplete synchronization patterns. As a result, the C value was 6.933.

We examined statistical significance from two perspectives. One was to clarify the difference between high- and low-level groups from the occurrence frequencies of the three patterns. To clarify the difference between groups, we permutated the observed pattern data into two groups; that is, the occurrence frequencies for the three patterns observed by combining high- with low-level groups, 230 s (R), 200 s (PA), and 117 s (PI) , were randomly assigned to either the high- or low-level group and a C value was calculated for this permutated pattern. We conducted 5,000 rounds of permutation, and obtained the probability when permutated values were greater than an observed C value ($C = 6.933$, as above). Moreover, we repeated this procedure 30 times. As a result, we obtained the average of the probabilities, $p = 0.018$. This result shows there was a significant difference between groups in the occurrence frequencies of the three patterns.

Another was to examine the effect of subgroups in each level. Because we had four subgroups in each level, the occurrence frequencies for three patterns were calculated in each subgroup. Then eight subgroups were assigned either to high- or low-level groups, and a C value was calculated for these permuted groups, repeating this procedure for all possibilities. The probability was 0.058 when the permutated C values were greater than the observed C value ($C = 6.933$, as above). This result also shows a difference between high- and low-level groups by significant tendency.

References

1. Edgington ES (1995) Randomization test: third edition, revised and expanded. New York: Marcel Dekker, Inc.
